# A Semi-quantitative Food Frequency Questionnaire Has Relative Validity to Identify Groups of NOVA Food Classification System Among Mexican Adults

**DOI:** 10.3389/fnut.2022.737432

**Published:** 2022-02-03

**Authors:** Cecilia Isabel Oviedo-Solís, Eric A. Monterrubio-Flores, Sonia Rodríguez-Ramírez, Gustavo Cediel, Edgar Denova-Gutiérrez, Simón Barquera

**Affiliations:** ^1^Center for Nutrition and Health Research, National Institute of Public Health, Cuernavaca, Mexico; ^2^School of Nutrition and Dietetics, University of Antioquia, Medellín, Colombia

**Keywords:** food frequency questionnaire, relative validity, 24 h dietary recall, NOVA food classification system, adult population

## Abstract

**Background:**

Ultra-processed foods are recognized as indicators of an unhealthy diet in epidemiological studies. In addition to ultra-processed foods, the NOVA food classification system identifies three other groups with less processing. Unprocessed foods that, together with minimally processed foods (MPF), make NOVA group 1, NOVA group 2 is processed culinary ingredients, and NOVA group 3 is processed foods.

**Objective:**

To assess the relative validity of the semi-quantitative food frequency questionnaire (SFFQ) to estimate the energy intake for each group NOVA classification system by comparing it with two 24 h-dietary-recall (24DRs) Mexican adults.

**Methods:**

We analyzed dietary information from 226 adults included <60 and ≥60 years with complete SFFQ and two 24DRs from the National Health and Nutrition Survey 2012. We reported mean differences, Spearman correlation coefficients, intra-class correlations coefficients, Bland–Altman plots, and weighted kappa between measures.

**Results:**

The percentage energy intake from unprocessed and minimally processed foods group, Spearman correlation coefficients was 0.54 in adults <60 years and 0.42 in adults ≥60 years, while ultra-processed foods group was 0.67 and 0.48, respectively. The intra-class correlation coefficients in the unprocessed and minimally processed foods group was 0.51 in adults <60 years and 0.46 in adults ≥60 years, and for the ultra-processed foods group were 0.71 and 0.50, respectively. Bland–Altman plots indicated reasonably consistent agreement for unprocessed and minimally processed foods group and ultra-processed foods group in adults <60 years and adults in the ≥60 age group. Weighted kappa was 0.45 in the ultra-processed foods group to adults <60 years and was 0.36–≥60 years.

**Conclusion:**

The SFFQ had acceptable validity to rank the percentage of energy intake from unprocessed and minimally processed foods group and ultra-processed foods group in Mexican adults, both in adults under 60 years and who were 60 years old or older.

## Introduction

In epidemiological studies, ultra-processed foods are recognized as an unhealthy diet indicator (1–12). As defined by the NOVA food classification system (belonging to NOVA group 4), ultra-processed foods are industrial formulations of food substances often modified by a chemical process and frequent use of cosmetic additives and sophisticated packaging (13). In adults, high consumption of ultra-processed food is associated with adverse health outcomes (14–16).

In addition to ultra-processed foods, the NOVA food classification system identifies three other groups with a lesser degree of processing. Unprocessed and minimally processed foods make up NOVA group 1, NOVA group 2 is processed culinary ingredients, and NOVA group 3 is processed foods (13).

Instruments such as 24-h recall (24DR) or dietary records have been used to measure food intake for each NOVA classification group (15). These are open-ended, including very detailed information that can collect information on the type of processing (14, 17). However, in studies with a large population, these methods are generally expensive, unrepresentative of usual intake if only a few days are assessed and inappropriate for evaluating past diet (18).

The instrument designed to capture habitual intake is the semi-quantitative food frequency questionnaire (SFFQ). This instrument has benefits like low cost and is the most useful for epidemiological studies as case-control and cohort studies and nationally representative surveys (19). Nevertheless, its potential to provide information depends on the level of detail of the food list (20).

The SFFQ used in national surveys in Mexico was designed to assess the relationship between dietary intake and chronic diseases in epidemiological studies (21, 22). Since 2006, the SFFQ has provided information about population nutrition (23) and evaluated the impact of policies and programs related to nutrition (24).

The SFFQ used in a national survey in 2012 is an adapted version of the questionnaire employed in 2006 (25). This questionnaire has been evaluated to assess food intake (26) and dietary patterns (27). However, until now, the SFFQ do not has been evaluated to identify NOVA food groups. Therefore, understanding the validity and magnitude of the error with the SFFQ to identify NOVA food groups allows us to interpret the SFFQ dietary information results and monitor changes in consumption of ultra-processed food or other NOVA groups in the Mexican population.

Therefore, we aim to assess the relative validity of the SFFQ for estimating the energy intake for each group NOVA classification system by comparing it with 24DRs in Mexican adults.

## Materials and Methods

### Study Design and Participants

The diet information for the present study was obtained from the Mexican National Health Nutrition Survey 2012 (ENSANUT 2012, by its Spanish acronym). The ENSANUT 2012 is a nationally representative survey, probabilistic with multi-stage stratified sampling. The coverage and methodology design of the national survey was previously published (28). ENSANUT 2012 obtained information of 46,303 adults (≥20 years). The collection of the ENSANUT 2012 data was carried out between October 2011 and May 2012. Detailed dietary information was collected from a random subsample. Adults with complete information of one SFFQ and two 24DRs were 252 of them, 178 adults <60 years and 80 adults ≥60 years. Among them, 10.3% (*n* = 26) of participants were excluded from the analysis because diet energy or food intake was potentially implausible. Therefore, our final analysis included 226 participants, 158 adults <60 years and 68 ≥ 60 years.

The survey protocol (CI: 1033) and the secondary analysis were approved by the Research, Biosafety, and Ethics Committees of the National Public Health Institute in Cuernavaca, Mexico, and have therefore been performed by the ethical standards laid down in the 1964 Declaration of Helsinki and its later amendments. In the National Health and Nutrition Survey 2012, an informed consent format was implemented for all participants. For the present analysis, only anonymized data were used.

### Dietary Assessment

#### Semi-Quantitative Food Frequency Questionnaire

One SFFQ was administered by trained health personnel through in-person interviews with the software (Visual FoxPro program, v.7) designed specifically for ENSANUT-2012 (25). The SFFQ included the consumption of 140 food items, and the interviewers asked study subjects to recall all foods and portions consumed in the seven days before the interview. For estimations, the number of days was multiplied by the number of times per day that the food item was consumed in the last seven days, and then the portion size per day was calculated. The daily frequency of consumption (portions/day) of each food was multiplied by the energy content of the food to calculate the energy consumption (kcal/day) using the food composition tables (29) compiled by the Institute of Public Health in Mexico.

#### Twenty Four-Hour Dietary Recall

Two 24DRs were administered by personnel trained in standardized methods collected all the required information through in-person interviews using the automated 5-step multiple-pass method software adapted for the Mexican population (24DR-AMPM software version 1.0) (27). This method uses five iterative steps that complement each other to improve memory about food intake and reduce underreporting (30). The 24DRs were administered on a randomly selected day of the week, with approximately 50% obtained on weekend days (31). The first 24DR was obtained on the same day as the SFFQ (27), and the second was obtained on non-consecutive days. The mean number of days between the first and second 24DR was 2.4 ± 1.2 days (31). Participants were asked in detail about their food consumption during the previous day. At the beginning of the interview, participants listed the foods they had consumed during the previous day. Afterward, the interviewer returned to the preliminary food list and helped the interviewer remember frequently omitted foods. Subsequently, the list of foods was organized according to the moment and context in which each food was consumed. Then, detailed information about each food was collected. Finally, a review of the final food list was performed to obtain additional information or to correct any specific information that was incorrectly registered. The extended description of multiple pass 24DR was previously reported (30).

### Food Classification

The foods and beverages reported in the two 24DRs and an SFFQ were classified according to the extent and purpose of food processing with the NOVA classification system (Supplementary Figure 1). The foods were classified as (a) unprocessed and minimally processed foods group (e.g., fruits, vegetables, fresh meat, legumes, *tortilla*); (b) processed culinary ingredients group (e.g., plant oils, sugar, animal fats); (c) processed foods group (e.g., unpackaged fresh bread, cheese, salted meat, preserved vegetables, and fruits); (d) ultra-processed foods group (e.g., carbonated soft drinks, sweet snacks, confectionery, reconstituted meat products, industrial packaged bread, ready-to-eat products like “nuggets”, and “sticks”). Additionally, we analyzed unprocessed and minimally processed foods group and processed culinary ingredients jointly, considering they are frequently used together in culinary preparations.

### Sociodemographic Characteristics

Sociodemographic characteristics such as age and sex were obtained with predefined questionnaires. The socioeconomic level was calculated using the principal components method using the information on wellbeing for the entire population, including the home and domestic appliances possession. The index obtained was divided into tertiles that represented a low, medium, and high socioeconomic level. Localities with <2,500 residents were considered rural, and those with 2,500 or more residents were considered urban.

### Statistical Analysis

To clean participants' dietary data, we excluded those who had consumed more than three foods above three standard deviations (3 SD) in grams from the analysis. And then, the extreme upper values of energy intake with ratios above +3 SD of energy intake/estimated energy requirement (EER). We estimate EER from the Institute of Medicine (IOM) equations (32). Moreover, to clean data at the lower extreme energy intake values, we excluded subjects with energy intake/basal metabolic rate (BMR) ratios below 0.5. According to the Mifflin-St Jeor equations (33), the BMR was estimated, as indicated in the methodological report (25). We calculated the average of both 24DRs for each person. Then, we estimated % of energy intake and energy intake for 24DRs and SFFQ by NOVA categories.

To assess relative validity, we compared SFFQ against the 24DRs for % energy intake and energy intake (kcal) for all four of the NOVA categories and unprocessed and minimally processed foods group and processed culinary ingredients group jointly. We calculated mean, confidence intervals, and median, Q1–Q3, and reported significance with paired *t*-tests (parametric distribution) or Wilcoxon signed-rank test (non-parametric distribution), spearman correlations, and intra-class correlations between measures. We generated Bland–Altman plots with limits of agreement (95%).

We categorized energy intake by quintiles to assess the ranking ability of the SFFQ, as the proportion of participants who were correctly classified (same quintile), adjacently classified (same or next quintile), or grossly misclassified (highest quintile by SFFQ and lowest by 24DRs, or vice versa).

To interpret the strength of agreement, Landis and Koch (34) suggest the following interpretations for below 0.0 poor, 0.00–0.20 slight, 0.21–0.40 fair, 0.41–0.60 moderate, 0.61–0.80 substantial, 0.81–1.00 almost perfect.

Finally, we calculated weighted kappa to eliminate the random effect. Significance was considered when *P* < 0.05. The Stata statistical software version 14.0 was used for all analyses.

## Results

We analyzed dietary information from 226 adults; 70% (*n* = 158) were <60 years old the mean age was 38.9, 95% CI: 37.2, 40.5. In older adults (≥60 years), the mean age was 72.1, 95% CI: 70.3, 74.0 years. A higher proportion of participants were women (56.6%). In older adults (≥60 years), the proportion of men and women was equal (50%). Most were from urban areas (62.8%), and only 3.1% were from Mexico City (Table 1).

**Table 1:** Characteristics of the participants in the relative validation study.

	**Total adults**	**Adults (<60 y)**	**Older adults (≥60 y)**
	***n* = 226**	***n* = 158**	***n* = 68**
**Age, mean (95% CI)**	48.9 (46.5, 51.2)	38.9 (37.2, 40.5)	72.1 (70.3, 74.0)
**Sex, % (95% CI)**
Men	43.4 (37.0, 50.0)	40.5 (33.1, 48.4)	50.0 (38.2, 61.8)
Women	56.6 (50.0, 63.0)	59.5 (51.6, 66.9)	50.0 (38.2, 61.8)
**Area^a^, % (95% CI)**			
Urban	62.8 (56.3, 68.9)	64.6 (56.7, 71.7)	58.8 (46.7, 70.0)
Rural	37.2 (31.1, 43.7)	35.4 (28.3, 43.3)	41.2 (30.0, 53.3)
**Socioeconomic level^b^, % (95% CI)**
Tertile 1	36.7 (30.7, 43.3)	36.1 (28.9, 43.9)	38.2 (27.4, 50.4)
Tertile 2	36.3 (30.2, 42.8)	35.4 (28.3, 43.3)	38.2 (27.4, 50.4)
Tertile 3	27.0 (21.6, 33.2)	28.5 (21.9, 36.1)	23.5 (14.9, 35.2)
**Region, % (95% CI)**
North	24.3 (19.1, 30.4)	24.1 (18.0, 31.4)	25.0 (16, 36.8)
Central	31.9 (26.1, 38.3)	29.7 (23.1, 37.4)	36.8 (26.1, 48.9)
Mexico city	3.1 (1.5, 6.4)	3.2 (1.3, 7.4)	2.9 (0.7, 11.2)
South	40.7 (34.4, 47.3)	43.0 (35.5, 50.9)	35.3 (24.8, 47.4)

a *Area rural defined by rural localities with <2,500 habitants, and urban with 2,500 or more*.

b *Tertiles of the socioeconomic index based on household characteristics using the information on well-being and domestic appliances possession*.

Table 2 shows the mean of total energy intake by 24DRs was 1,765.0, 95% CI: 1,682.8, 1,847.2 K_cal_, while in SFFQ was 1,793.2, 95% CI: 1,696.9, 1,889.4 K_cal_. No significant differences in total energy intake by dietary assessment instruments were shown. However, the percentage of energy between SFFQ and 24DRs by NOVA groups were significantly different in the unprocessed and minimally processed foods group, processed culinary ingredients group, and processed foods group. The SFFQ underestimated the processed culinary ingredients group (%K_cal_ = −6.4 95% CI = −7.1, −5.6). On the other hand, the SFFQ overestimated the unprocessed and minimally processed foods group (%K_cal_ = 4.2, 95% CI = 2.1, 6.4) and processed foods group (%K_cal_ = 1.7, 95% CI = 0.3, 3.2). The SFFQ overestimated energy intake (K_cal_) unprocessed and minimally processed foods group and underestimated the processed culinary ingredients group (Supplementary Table 1).

**Table 2:** Percentage energy intakes and difference mean between SFFQ and 24DRs in adults and older adults in NOVA foods groups.

**NOVA foods groups**	**24DRs**			**SFFQ**			**Mean difference^a^ (95% CI) between SFFQ and 24DRs**		
	**Total adults**	**Adults (<60 y)**	**Older adults (≥60 y)**	**Total adults**	**Adults (<60 y)**	**Older adults (≥60 y)**	**Total adults**	**Adults (<60 y)**	**Older adults (≥60 y)**
Total energy intake [kcal], mean (95% CI)	1,765.0 (1,682.8, 1,847.2)	1,842.9 (1,745.1, 1,940.7)	1,584.0 (1,437.6, 1,730.4)	1,793.2 (1,696.9, 1,889.4)	1,911.0 (1,791.6, 2,030.4)	1,519.5 (1,376.6, 1,662.4)	28.1 (−63.5, 119.8)	68 (−47.3, 183.4)	−64.5 (−211.3, 82.2)
Total energy intake [kcal], median (Q1,Q3)	17,29.7 (1,254.8, 2,180.7)	1,859.1 (1,336.4, 2,266.2)	1,516.4 (1,133.5, 1,982.2)	1,685.2 (1,270.2, 2,240.1)	1,778.4 (1,306.2, 2,350)	1,414.7 (1,172.1, 1,773.8)			
**Unprocessed and minimally processed foods group**								
Energy intake percentage, mean (95% CI)	60.2 (57.9, 62.5)	58.1 (55.3, 60.9)	65 (60.9, 69.1)	64.4 (62.4, 66.4)	63.4 (61, 65.8)	66.9 (63.4, 70.4)	4.2 (2.1, 6.4)^c^	5.2 (2.7, 7.8)^c^	1.9 (−2, 5.9)
Energy intake percentage, median (Q1,Q3)	60.7 (47.8, 73)	59.1 (45.4, 70.1)	62.4 (54.1, 80)	64.2 (55, 74.9)	63 (54.6, 73.7)	68.6 (57.7, 77.6)			
**Processed culinary ingredients group**								
Energy intake percentage, mean (95% CI)	9.1 (8.3, 9.9)	9.1 (8.3, 10)	9 (7.3, 10.6)	2.7 (2.3, 3.1)	2.5 (2.1, 2.9)	3.3 (2.3, 4.2)	−6.4 (−7.1, −5.6)^c^	−6.7 (−7.6, −5.8)^c^	−5.7 (−7.2, −4.2)^c^
Energy intake percentage, median (Q1,Q3)	8 (4.8, 12.4)	8 (4.8, 12.6)	7.5 (4.3, 12.1)	2 (0.3, 4)	1.9 (0.5, 3.8)	2.8 (0.1, 4.7)			
**Processed foods group**								
Energy intake percentage, mean (95% CI)	11.5 (10.1, 12.9)	11.4 (9.7, 13)	11.9 (9.1, 14.7)	13.2 (12.1, 14.4)	13.3 (11.9, 14.7)	13.2 (11, 15.4)	1.7 (0.3, 3.2)^c^	1.9 (0.1, 3.7)^c^	1.3 (−1.3, 4)
Energy intake percentage, median (Q1,Q3)	9.4 (1.8, 17.2)	9.4 (2.6, 16.2)	9.1 (1.6, 18.7)	12.7 (6.1, 18.3)	12.6 (6.5, 17.8)	12.8 (5.6, 20.1)			
**Ultra-Processed foods group**								
Energy intake percentage, mean (95% CI)	19.2 (17.1, 21.3)	21.4 (18.8, 24)	14.2 (10.7, 17.6)	19.6 (17.7, 21.5)	20.9 (18.5, 23.2)	16.6 (13.6, 19.7)	0.4 (−1.2, 2)	−0.5 (−2.4, 1.4)	2.4 (−0.8, 5.7)
Energy intake percentage, median (Q1,Q3)	15.7 (5.9, 30)	18.2 (8, 32.8)	8.9 (1.8, 21.9)	17.9 (7.6, 28.3)	20.8 (8.7, 29.8)	13.6 (6.4, 25.6)			
**Unprocessed and minimally processed foods group and processed culinary ingredients group**								
Energy intake percentage, mean (95% CI)	69.3 (66.9, 71.6)	67.3 (64.4, 70.1)	73.9 (69.9, 78)	67.2 (65.1, 69.2)	65.8 (63.4, 68.3)	70.2 (66.7, 73.7)	−2.1 (−4.2, −0.1)^c^	−1.4 (−3.8, 1)	−3.8 (−7.5, 0)^b^
Energy intake percentage, median (Q1,Q3)	69.7 (56.6, 84.1)	68.1 (54.2, 81)	71.2 (63.5, 88.6)	67.9 (58.1, 77.7)	65.3 (55.6, 75.9)	72.4 (61.3, 80.7)			

a *Difference (24DRs-SFFQ)*.

b *Significance (P < 0.05) by paired t-tests*.

c *Significance (P < 0.05) by Wilcoxon signed-rank test*.

Spearman correlation and intra-class correlation coefficients between the SFFQ and 24DRs in adults were substantial for percentage energy intake from ultra-processed foods group (*r* = 0.62, ICC = 0.67), and moderate for unprocessed and minimally processed foods group (*r* = 0.52, ICC = 0.50) and unprocessed and minimally processed foods group and processed culinary ingredients group jointly (*r* = 0.56, ICC = 0.57), but showed poor agreement for processed culinary ingredients group (*r* = 0.24, ICC = 0.0) and fair for processed foods group (*r* = 0.37, ICC = 0.35) (Table 3). The Spearman correlation and intraclass correlation coefficient had higher values in adults <60 years in the unprocessed and minimally processed foods group and ultra-processed foods group. The percentage energy intake from unprocessed and minimally processed foods group Spearman correlation was 0.54 in adults <60 years and 0.42 in adults ≥60 years, while ultra-processed foods group was 0.67 and 0.48, respectively. The intra-class correlation coefficient in the unprocessed and minimally processed foods group was 0.51 in adults <60 years and 0.46 in adults ≥60 years, and for the ultra-processed foods group were 0.71 and 0.50, respectively (Table 3). Spearman correlation and Intra-class correlation coefficients between the SFFQ and 24DRs in adults <60 years were substantial for energy intake (K_cal_) from ultra-processed foods group with upper values than 0.60, while older adults, values were upper than 0.36. The unprocessed and minimally processed foods group were substantial for energy intake (K_cal_) in Spearman and Intra-class correlation coefficients in older adults, while adults <60 years, values were upper than 0.36 (Supplementary Table 2).

**Table 3:** Correlation between SFFQ and 24DRs in adults and older adults in NOVA foods groups.

**NOVA foods groups**	**Spearman correlation coefficient (95% CI) between SFFQ and 24DRs**			**Intra-Class correlation coefficient (95% CI) between SFFQ and 24DRs**		
	**Total adults**	**Adults (<60 y)**	**Older adults (≥60 y)**	**Total adults**	**Adults (<60 y)**	**Older adults (≥60 y)**
**Total energy intake (kcal)**	0.49 (0.38, 0.58)	0.48 (0.35, 0.59)	0.46 (0.25, 0.63)	0.48 (0.38, 0.58)	0.44 (0.31, 0.57)	0.49 (0.30, 0.67)
**Unprocessed and minimally processed foods group**						
Energy intake percentage	0.52 (0.42, 0.61)	0.54 (0.42, 0.64)	0.42 (0.20, 0.60)	0.50 (0.4, 0.6)	0.51 (0.39, 0.62)	0.46 (0.27, 0.65)
**Processed culinary ingredients group**						
Energy intake percentage	0.24 (0.11, 0.36)	0.22 (0.06, 0.36)	0.28 (0.05, 0.49)	0 (0, 0.13)	0 (0, 0.16)	0.09 (0, 0.32)
**Processed foods group**						
Energy intake percentage	0.37 (0.25, 0.47)	0.32 (0.17, 0.46)	0.44 (0.22, 0.61)	0.35 (0.24, 0.47)	0.31 (0.17, 0.45)	0.44 (0.25, 0.63)
**Ultra-processed foods group**						
Energy intake percentage	0.62 (0.54, 0.7)	0.67 (0.58, 0.75)	0.48 (0.27, 0.64)	0.67 (0.6, 0.74)	0.71 (0.63, 0.79)	0.50 (0.32, 0.68)
**Unprocessed and minimally processed foods group and processed culinary ingredients group**						
Energy intake percentage	0.56 (0.46, 0.64)	0.57 (0.45, 0.67)	0.47 (0.26, 0.64)	0.57 (0.48, 0.66)	0.58 (0.48, 0.69)	0.49 (0.31, 0.67)

**Figure 1:**
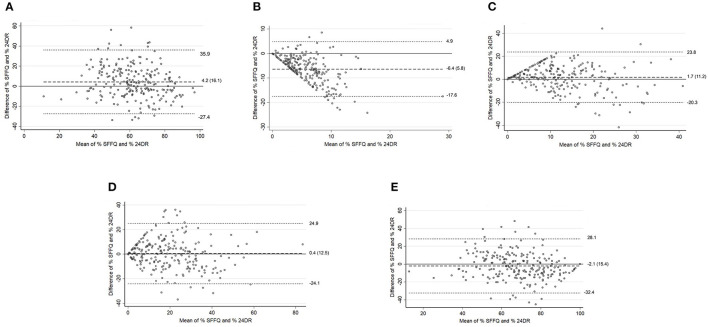
Total adults. Bland–Altman plots for % energy intake between the SFFQ and 24DRs: **(A)** Unprocessed and minimally processed foods group. **(B)** Processed culinary ingredients group. **(C)** Processed foods group. **(D)** Ultra-processed foods group. **(E)** Unprocessed and minimally processed foods group and processed culinary ingredients group. Dotted lines represent 95% limits of agreement. Dashed line represents difference of mean.

In total adults, Bland–Altman plots showed reasonable agreement in energy intake percentage between SFFQ and 24DRs for unprocessed and minimally processed foods group, ultra-processed foods group, and unprocessed and minimally processed foods group and processed culinary ingredients group jointly. The minimum bias between SFFQ and 24DRs was observed in the ultra-processed foods group (difference of mean = 0.4). The processed culinary ingredients group showed the poorest agreement (Figure 1). The Bland-Altman plots showed consistent agreement regarding energy intake (K_cal_) and energy intake percentage (Supplementary Figure 2).

**Figure 2:**
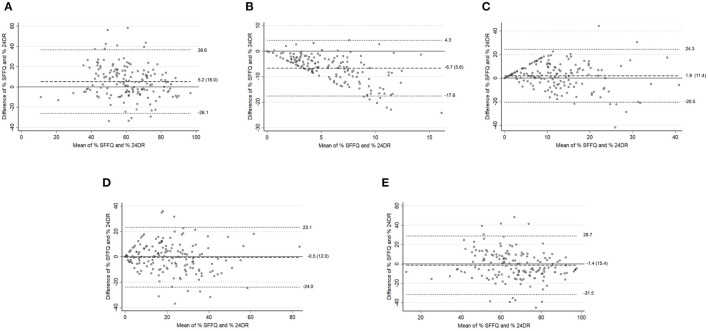
Adults (<60 y). Bland–Altman plots for % energy intake between the SFFQ and 24DRs: **(A)** Unprocessed and minimally processed foods group. **(B)** Processed culinary ingredients group. **(C)** Processed foods group. **(D)** Ultra-processed foods group. **(E)** Unprocessed and minimally processed foods group and processed culinary ingredients group. Dotted lines represent 95% limits of agreement. Dashed line represents difference of mean.

**Figure 3:**
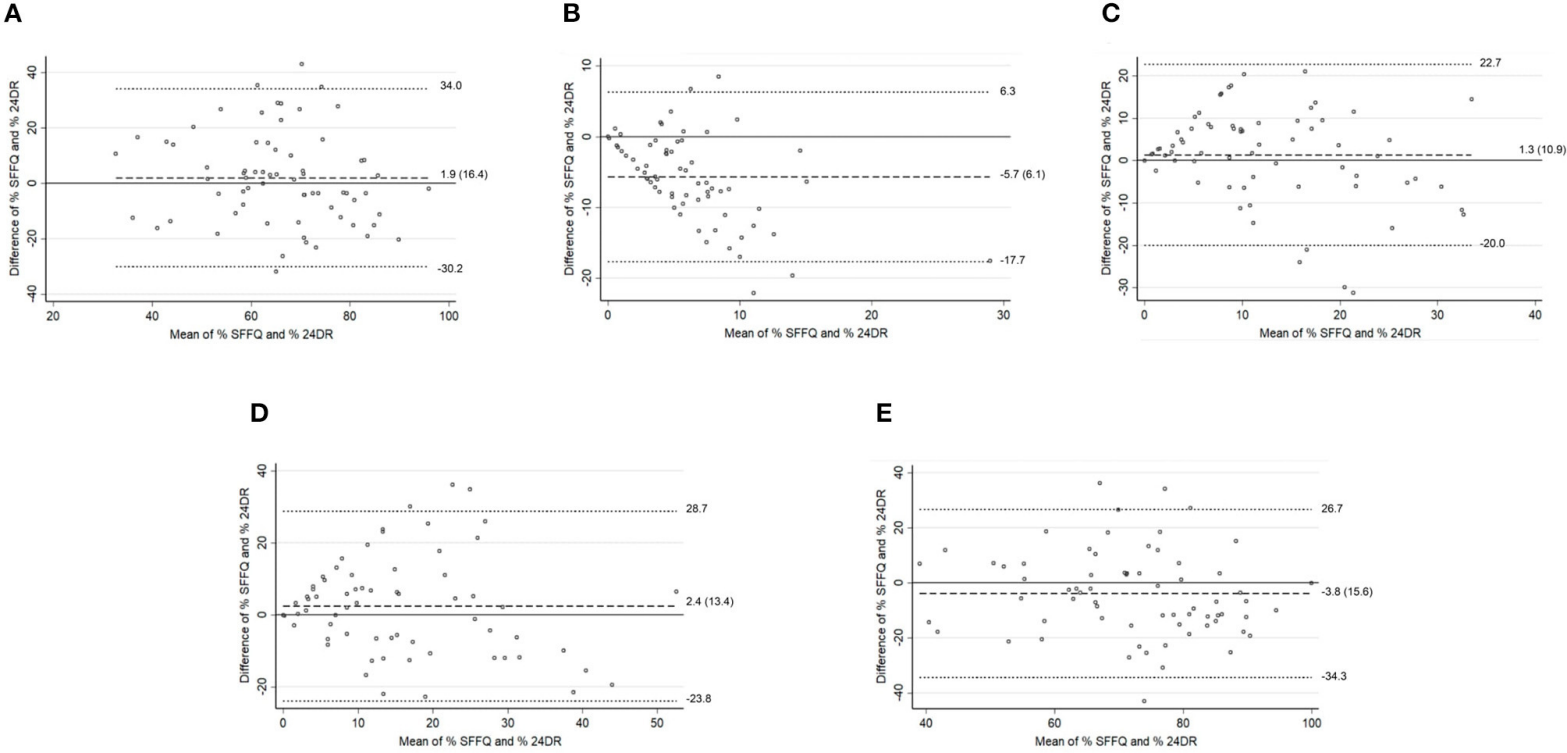
Older adults (≥60 y). Bland–Altman plots for % energy intake between the SFFQ and 24DRs: **(A)** Unprocessed and minimally processed foods group. **(B)** Processed culinary ingredients group. **(C)** Processed foods group. **(D)** Ultra-processed foods group. **(E)** Unprocessed and minimally processed foods group and processed culinary ingredients group. Dotted lines represent 95% limits of agreement. Dashed line represents difference of mean.

The Bland–Altman plots showed reasonable agreement in adults <60 years (Figure 2) in energy intake percentage between SFFQ and 24DRs for the unprocessed and minimally processed foods group and the ultra-processed foods group. The minimum bias between SFFQ and 24DRs was observed in the ultra-processed foods group (difference of mean = −0.5). The processed culinary ingredients group showed the poorest agreement. These results were consistent with Bland–Altman plots to energy intake (K_cal_) (Supplementary Figure 3). For adults in the ≥60 age group (Figure 3), Bland–Altman plots showed reasonable agreement in energy intake percentage between SFFQ and 24DRs for unprocessed and minimally processed foods group, ultra-processed foods group, and unprocessed and minimally processed foods group and processed culinary ingredients group jointly. The minimum bias between SFFQ and 24DRs was observed in the processed foods group and unprocessed and minimally processed foods group (difference of mean=1.3 and 1.9, respectively). The processed culinary ingredients group showed the poorest agreement. The Bland–Altman plots showed reasonable agreement in energy intake (K_cal_) between SFFQ and 24DRs for unprocessed and minimally processed foods group, and the minimum bias between SFFQ and 24DRs was observed in this group (Supplementary Figure 4).

Cross classification by quintiles of the percentage of energy consumption between SFFQ and 24DR in adults was correctly or adjacently classified in more than 70% of unprocessed and minimally processed foods group and ultra-processed foods group; the same was observed stratified groups of adults <60 years and adults ≥60 years. Furthermore, weighted kappa indicated acceptable agreement in percentage energy intake in unprocessed and minimally processed foods group and ultra-processed foods group (0.39, 0.43; respectively). In the adults <60 years, the agreement in the ultra-processed foods group was 0.45, but in the adults ≥60 years, it was 0.36 (Table 4). Cross classification by quintiles of energy intake (K_cal_) between SFFQ and 24DRs was superior to 60% to correctly or adjacently classified; weighted kappa showed acceptable agreement in energy intake (K_cal_) in ultra-processed foods group 0.47 in total adults, 0.51 in adults <60 y and finally, 0.31 in older adults (Supplementary Table 3).

**Table 4:** Cross classification by quintiles of energy consumption between SFFQ and 24DRs in NOVA foods groups.

**NOVA foods groups**	**Correctly classified (%)^a^**	**Correctly or adjacently classified (%)^b^**	**Grossly misclassified (%)^c^**	**Weighted kappa (SD)**
**Total adults**				
**Unprocessed and minimally processed foods group**				
Energy intake percentage	38.1	75.7	2.2	0.39 (0.05)^d^
**Processed culinary ingredients group**				
Energy intake percentage	29.2	59.3	6.2	0.17 (0.05)^d^
**Processed foods group**				
Energy intake percentage	29.6	65.5	3.1	0.23 (0.05)^d^
**Ultra-processed foods group**				
Energy intake percentage	37.2	78.8	1.8	0.43 (0.05)^d^
**Unprocessed and minimally processed foods group and processed culinary ingredients group**				
Energy intake percentage	38.9	78.3	2.2	0.43 (0.05)^d^
**Adults (<60 y)**				
**Unprocessed and minimally processed foods group**				
Energy intake percentage	38.0	77.2	1.9	0.40 (0.05)^d^
**Processed culinary ingredients group**				
Energy intake percentage	28.5	59.5	4.4	0.16 (0.05)^d^
**Processed foods group**				
Energy intake percentage	29.1	63.3	3.2	0.21 (0.05)^d^
**Ultra-processed foods group**				
Energy intake percentage	38.0	80.4	1.9	0.45 (0.05)^d^
**Unprocessed and minimally processed foods group and processed culinary ingredients group**				
Energy intake percentage	43.0	80.4	1.9	0.46 (0.05)^d^
**Older adults (≥60 y)**				
**Unprocessed and minimally processed foods group**				
Energy intake percentage	38.2	72.1	2.9	0.34 (0.08)^d^
**Processed culinary ingredients group**				
Energy intake percentage	30.9	58.8	10.3	0.20 (0.09)^d^
**Processed foods group**				
Energy intake percentage	30.9	70.6	2.9	0.28 (0.09)^d^
**Ultra-processed foods group**				
Energy intake percentage	35.3	75.0	1.5	0.36 (0.08)^d^
**Unprocessed and minimally processed foods group and processed culinary ingredients group**				
Energy intake percentage	29.4	73.5	2.9	0.32 (0.08)^d^

a *Correctly classified = % of adolescents with 24DRs and SFFQ intakes in the same quintile*.

b *Correctly or adjacently classified = % of adolescents with 24DRs and SFFQ intakes in the same or adjacent quintile*.

c *Grossly misclassified = % of adolescents with 24DRs intakes in the highest quintile and SFFQ intakes in the lowest quintile, or vice versa*.

d *Significance (P < 0.05)*.

## Discussion

In general, we show that the SFFQ has relative validity for ranking the percentage of energy intake from unprocessed and minimally processed foods group and ultra-processed foods group in Mexican adults. The SFFQ is not able to estimate energy intake or percentage energy intake from group processed culinary ingredients. Furthermore, the SFFQ showed acceptable agreement in adults ≥60 years. In previous studies, diet validation, the value of ≈0.4 has been considered an acceptable agreement (35).

Until now, we have not come across other reports that explore the relative validity of SFFQ to identify consumption of ultra-processed food or other groups of NOVA in adults. However, our results were consistent with the previous study in New Zealand children (≥5.0– ≤ 6.0 years) that showed acceptable relative validity for percentage energy intake from unprocessed and minimally processed foods group (ICC=0.31) and ultra-processed foods group (ICC = 0.30) using an SFFQ named EAT5 FFQ (17).

The lack of agreement showed the SFFQ vs. 24DRs from group processed culinary ingredients and group processed foods can be explained by the limited list of food items (25), which does not allow more detail of specific foods or culinary preparations; this is especially important to detect processed culinary ingredients. The unprocessed and minimally processed foods group and processed culinary ingredients group are used jointly to culinary preparations. Our results showed acceptable agreement when considered unprocessed and minimally processed foods group and processed culinary ingredients group jointly.

On the other hand, the SFFQ evaluated in the present study is unusual in frame time compared to other semi-quantitative food frequency questionnaires because it is generally used last year to collect information from the usual diet (36). Therefore, we assume a low week-week variability of the diet to estimate the usual diet. However, by asking for the last week, it is possible to improve the accuracy of the information and reduce recall error (19). This aspect is crucial in older adults.

The SFFQ sacrifices precise intake measurements, but at the same time, it may better represent long-term or average feeding behavior. Studies in cognitive research report that it is easier to describe the foods one usually consumes (generic memory) rather than describe what foods were eaten at any specific meal in the past (episodic memory) (36). Although SFFQ and 24DR are based on memory, for the elderly, simple well-conducted methods (24DR and SFFQ) for assessing group mean dietary intakes may give more accurate information than methods not based on memory (the dietary record) (37, 38).

However, there is no current evidence that older adults provide less valid self-reports using methods such as SFFQ or 24DR, compared with younger adults (39, 40). Our results showed that SFFQ has an acceptable agreement in adults <60 years and those ≥60 years.

In the present study, some limitations must be considered. First, there could be a correlation of errors between the two instruments due to the temporal relationship and the reference frame in their application (19) and respondent fatigue errors. Second, it is likely that some foods between the NOVA groups were misclassified. In less than 10% of cases, the 24DR did not have a list of all ingredients used in culinary preparations, which was reported as “standard preparation.” In these cases, the principal ingredient of preparation was classified in one NOVA group, just as culinary preparation was classified in the SFFQ. Moreover, if it was unclear whether food products were homemade or commercially manufactured, the less processed category was chosen when in doubt as the previous report (41).

Finally, in the ENSANUT 2012, only 7% of older adults had a cognitive impairment, so that the diet information was answered by a person in charge of feeding them or by their caretaker (42). Both recall and food frequency techniques are inappropriate if memory or cognitive functioning is impaired (43). However, we considered it necessary to evaluate this age group to test the instrument's ability to accurately estimate ultra-processed foods group consumption, given possible implications of adverse health outcomes.

The strengths of our study include that SFFQ was specially designed for use in the Mexican population, and the sample size was adequate for a validation study and was obtained from a national survey. The 24DRs were obtained on non-consecutive days as recommended since eating habits from consecutive days have been shown to be correlated (44). On the other hand, personnel was trained and standardized to collect all the required information through face-to-face interviews to ensure the quality of the information (28).

These results, coming from a representative sample, are the first of this type in Mexico and Latin America in general, which can serve as a baseline to carry out other studies of a longitudinal nature, evaluating the effects of ultra-processed foods group consumption using the SFFQ. Ultra-processed foods consumption is one of the trending topics due to its relationship with chronic illnesses and new politics in public health (45).

In conclusion, the SFFQ had acceptable validity to rank the percentage of energy intake from unprocessed and minimally processed foods group and ultra-processed foods group in Mexican adults, both in adults under 60 years and who were 60 years old or older. However, the SFFQ was not recommended to identify processed culinary ingredients group according to the NOVA classification system.

## Data Availability Statement

The raw data supporting the conclusions of this article will be made available by the authors, without undue reservation.

## Ethics Statement

The studies involving human participants were reviewed and approved by Comité de Investigación del Instituto Nacional de Salud Pública. The patients/participants provided their written informed consent to participate in this study.

## Author Contributions

CIO-S developed research questions, analyzed and interpreted the data, and wrote the first draft of the manuscript. EAM-F and SR-R contributed data collection and support analyzed the data. GC, ED-G, and SB support interpreted the data and assisted in the writing of the manuscript. All authors read and approved the final manuscript before submission.

## Funding

This work was carried during the period from 2017 to 2021. CIO-S received a grant from CONACyT to study for a Ph.D. degree (589934).

## Conflict of Interest

The authors declare that the research was conducted in the absence of any commercial or financial relationships that could be construed as a potential conflict of interest.

## Publisher's Note

All claims expressed in this article are solely those of the authors and do not necessarily represent those of their affiliated organizations, or those of the publisher, the editors and the reviewers. Any product that may be evaluated in this article, or claim that may be made by its manufacturer, is not guaranteed or endorsed by the publisher.

## Abbreviations

24DR: 24 hour-dietary-recall
ENSANUT 2012: Mexican National Health Nutrition Survey 2012
SFFQ: Semiquantitative food frequency questionnaire.
