# Supplementary material for: A Semi-quantitative Food Frequency Questionnaire Has Relative Validity to Identify Groups of NOVA Food Classification System Among Mexican Adults
Source: Front Nutr. 2022 Feb 3;9:737432. doi: 10.3389/fnut.2022.737432 (PMC8850985; doi:10.3389/fnut.2022.737432)
Supplement: Supplementary file 1 [file Table_1.docx]

Supplementary Material

| **Supplementary table 1. Energy intakes and difference mean between SFFQ and 24DRs in adults and older adults in NOVA foods groups** | | | | | | | | | |
| --- | --- | --- | --- | --- | --- | --- | --- | --- | --- |
| **NOVA foods groups** | **24DRs** | | | **SFFQ** | | | **Mean difference (95% CI) between SFFQ and 24DRs** | | |
|  | **Total adultos** | **Adults (<60 y)** | **Older adults (≥60 y)** | **Total adultos** | **Adults (<60 y)** | **Older adults (≥60 y)** | **Total adultos** | **Adults (<60 y)** | **Older adults (≥60 y)** |
| **Unprocessed and minimally processed foods group** | | | | | | | | | |
| Energy intake [Kcal],mean (95% CI) | 1035.2 (980.8, 1089.6) | 1040 (977.9, 1102.1) | 1024.1 (912.5, 1135.7) | 1130 (1067.3, 1192.7) | 1180 (1104.2, 1255.8) | 1013.9 (904.5, 1123.2) | 94.8 (31.9, 157.7) ^c^ | 140 (63.7, 216.3) ^c^ | -10.2 (-119.7, 99.2) |
| Energy intake porcentage, median (Q1,Q3) | 1012.5 (739.1, 1282.1) | 1023.5 (756.3, 1288.3) | 947.7 (715.9, 1224.1) | 1037.7 (780.5, 1441.4) | 1139.8 (843.5, 1484.2) | 961 (662.8, 1341) |  |  |  |
|  |  |  |  |  |  |  |  |  |  |
| **Processed culinary ingredients group** | | | | | | | | | |
| Energy intake [Kcal],mean (95% CI) | 164 (147.6, 180.3) | 173.2 (153, 193.3) | 142.6 (115.1, 170.2) | 45.9 (39.4, 52.3) | 46 (38.5, 53.5) | 45.4 (32.8, 58.1) | -118.1 (-134.7, -101.5) ^c^ | -127.1 (-147.5, -106.8) ^c^ | -97.2 (-125.7, -68.6)^c^ |
| Energy intake porcentage, median (Q1,Q3) | 139.9 (70.1, 232.8) | 144 (74.6, 242.9) | 115.6 (62.1, 198) | 32.7 (4.7, 65.3) | 32.7 (7.8, 70) | 32.7 (1.2, 65.3) |  |  |  |
|  |  |  |  |  |  |  |  |  |  |
| **Processed foods group** | | | | | | | | | |
| Energy intake [Kcal],mean (95% CI) | 203.2 (176.1, 230.4) | 213.4 (179.4, 247.3) | 179.7 (135, 224.4) | 237.7 (212.7, 262.6) | 257.5 (225.3, 289.7) | 191.6 (157, 226.3) | 34.5 (4, 64.9) ^c^ | 44.1 (4.6, 83.7) ^c^ | 12 (-31.6, 55.5) |
| Energy intake porcentage, median (Q1,Q3) | 139.3 (27.5, 312) | 155 (37.5, 313.2) | 108.9 (20.9, 291.3) | 192.9 (92.9, 331.6) | 204.4 (111, 353.1) | 177.4 (85.1, 251) |  |  |  |
|  |  |  |  |  |  |  |  |  |  |
| **Ultra-processed foods group** | | | | | | | | | |
| Energy intake [Kcal],mean (95% CI) | 362.6 (314.2, 411) | 416.4 (355.6, 477.2) | 237.6 (167.1, 308) | 379.6 (331.4, 427.8) | 427.4 (364.8, 490.1) | 268.5 (207.1, 329.9) | 17 (-25.6, 59.6) | 11 (-41.1, 63.2) | 30.9 (-44.2, 106.1) |
| Energy intake porcentage, median (Q1,Q3) | 243.5 (99.7, 554.6) | 309.1 (125.7, 618.7) | 175.8 (21.5, 332.5) | 306.9 (107.3, 529.9) | 367.7 (129.5, 617.9) | 213.7 (76.6, 373.1) |  |  |  |
|  |  |  |  |  |  |  |  |  |  |
| **Unprocessed and minimally processed foods group and Processed culinary ingredients group** | | | | | | | | | |
| Energy intake [Kcal],mean (95% CI) | 1199.2 (1137.1, 1261.2) | 1213.2 (1140.9, 1285.4) | 1166.7 (1044.4, 1289.1) | 1175.9 (1112, 1239.7) | 1226 (1148.8, 1303.2) | 1059.3 (947.9, 1170.7) | -23.3 (-89.2, 42.6) | 12.9 (-67.6, 93.3) | -107.4 (-222, 7.2) |
| Energy intake porcentage, median (Q1,Q3) | 1149.3 (853.6, 1476.3) | 1157.4 (878.4, 1512.5) | 1126 (785.3, 1378.4) | 1085.8 (825.9, 1516) | 1163.7 (887.3, 1533.3) | 982.3 (699.1, 1376) |  |  |  |
| ^a^ Difference (24DRs- SFFQ) | | | | | | | | | |
| ^b^ Significance (P< 0.05) by paired t-tests | | | | | | | | | |
| ^c^ Significance (P< 0.05) by Wilcoxon signed-rank test | | | | | | | | | |
