# Supplementary material for: A Semi-quantitative Food Frequency Questionnaire Has Relative Validity to Identify Groups of NOVA Food Classification System Among Mexican Adults
Source: Front Nutr. 2022 Feb 3;9:737432. doi: 10.3389/fnut.2022.737432 (PMC8850985; doi:10.3389/fnut.2022.737432)
Supplement: Supplementary file 2 [file Table_2.docx]

Supplementary Material

| **Supplementary table 2. Correlation between SFFQ and 24DR in adults and older adults in NOVA foods groups** | | | | | | |
| --- | --- | --- | --- | --- | --- | --- |
| **NOVA foods groups** | **Spearman correlation coefficient (95% CI) between SFFQ and 24DRs** | | | **Intra-class correlation coefficient (95% CI) between SFFQ and 24DRs** | | |
|  | **Total adults** | **Adults (<60 y)** | **Older adults (≥60 y)** | **Total adults** | **Adults (<60 y)** | **Older adults (≥60 y)** |
| **Unprocessed and minimally processed foods group** | | | | | | |
| Energy intake [Kcal] | 0.44 (0.33, 0.54) | 0.41 (0.27, 0.53) | 0.50 (0.3, 0.66) | 0.41 (0.3, 0.52) | 0.36 (0.23, 0.5) | 0.51 (0.34, 0.69) |
|  |  |  |  |  |  |  |
| **Processed culinary ingredients group** | | | | | | |
| Energy intake [Kcal] | 0.21 (0.08, 0.33) | 0.2 (0.04, 0.34) | 0.22 (-0.02, 0.44) | 0 (0, 0.13) | 0 (0, 0.16) | 0 (0, 0.24) |
|  |  |  |  |  |  |  |
| **Processed foods group** | | | | | | |
| Energy intake [Kcal] | 0.38 (0.27, 0.49) | 0.36 (0.22, 0.49) | 0.41 (0.19, 0.59) | 0.31 (0.19, 0.43) | 0.27 (0.13, 0.42) | 0.41 (0.21, 0.61) |
|  |  |  |  |  |  |  |
| **Ultra-processed foods group** | | | | | | |
| Energy intake [Kcal] | 0.62 (0.53, 0.69) | 0.65 (0.55, 0.73) | 0.44 (0.22, 0.61) | 0.61 (0.53, 0.69) | 0.64 (0.55, 0.74) | 0.36 (0.15, 0.56) |
|  |  |  |  |  |  |  |
| **Unprocessed and minimally processed foods group and Processed culinary ingredients group** | | | | | | |
| Energy intake [Kcal] | 0.44 (0.33, 0.54) | 0.43 (0.29, 0.55) | 0.45 (0.24, 0.62) | 0.45 (0.35, 0.56) | 0.42 (0.3, 0.55) | 0.51 (0.33, 0.68) |
