# Supplementary material for: A Semi-quantitative Food Frequency Questionnaire Has Relative Validity to Identify Groups of NOVA Food Classification System Among Mexican Adults
Source: Front Nutr. 2022 Feb 3;9:737432. doi: 10.3389/fnut.2022.737432 (PMC8850985; doi:10.3389/fnut.2022.737432)
Supplement: Supplementary file 3 [file Image_1.pdf]

Supplementary Material

**Unprocessed or minimally processed foods group.**<br><br>Definition: Unprocessed, edible parts of plants or animals and fungi, algae, and water, after separation from nature. Minimally processed, unprocessed foods are altered by industrial processes such as removing inedible or unwanted parts, drying, boiling, pasteurization, refrigeration, freezing, non-alcoholic fermentation, and other methods that do not add salt, sugar, oils or fats, or other food substances to the original food.<br><br>Examples: Fresh, frozen, or dried fruits and vegetables; grains such as brown, or white rice; legumes such as beans and lentils; meat, poultry, and fish; eggs; fresh or pasteurized milk; herbs and spices, such as oregano, tea, coffee, and drinking water.
**Processed culinary ingredients group.**<br><br>Definition: Substances obtained directly from group MPF foods or nature by industrial processes such as pressing, centrifuging, refining, extracting, or mining. Their use is in the preparation, seasoning, and cooking of group MPF foods.<br><br>Examples: Vegetable oils, butter and lard obtained from milk and pork; sugar and molasses; honey and salt.
**Processed foods group.**<br><br>Definition: Products made by adding salt, oil, sugar, or other group PCI ingredients to group MPF foods, using preservation methods such as canning and bottling, and, in the case of bread and cheeses, using non-alcoholic fermentation.<br><br>Examples: Canned or bottled vegetables and legumes in brine; salted or sugared nuts and seeds; salted, dried, cured, or smoked meats and fish; canned fish; fruits in syrup; freshly made unpackaged breads and cheeses.
**Ultra-processed foods group.**<br><br>Definition: Formulations of ingredients, most exclusive industrial use, frequent whit application of additives whose function is to make the final product palatable or hyper-palatable. Ingredients often include sugar, oils and fats, and salt; substances that no or rare culinary use such as high fructose corn syrup, hydrogenated or interesterified oils, and protein isolates.<br><br>Examples: Carbonated soft drinks; sweet or savory packaged snacks; candies; ice-cream; mass-produced packaged bread and buns; margarine; cookies, cake mixes; breakfast ‘cereals’, ‘cereal’ and ‘energy’ bars; ‘energy’ drinks; ‘nuggets’ and ‘sticks,’ and packaged ‘instant’ soups, noodles and desserts.

Supplementary figure 1. Classification foods according to NOVA food groups, definitions, and examples.
