# Supplementary material for: A Semi-quantitative Food Frequency Questionnaire Has Relative Validity to Identify Groups of NOVA Food Classification System Among Mexican Adults
Source: Front Nutr. 2022 Feb 3;9:737432. doi: 10.3389/fnut.2022.737432 (PMC8850985; doi:10.3389/fnut.2022.737432)
Supplement: Supplementary file 6 [file Image_4.pdf]

Supplementary Material

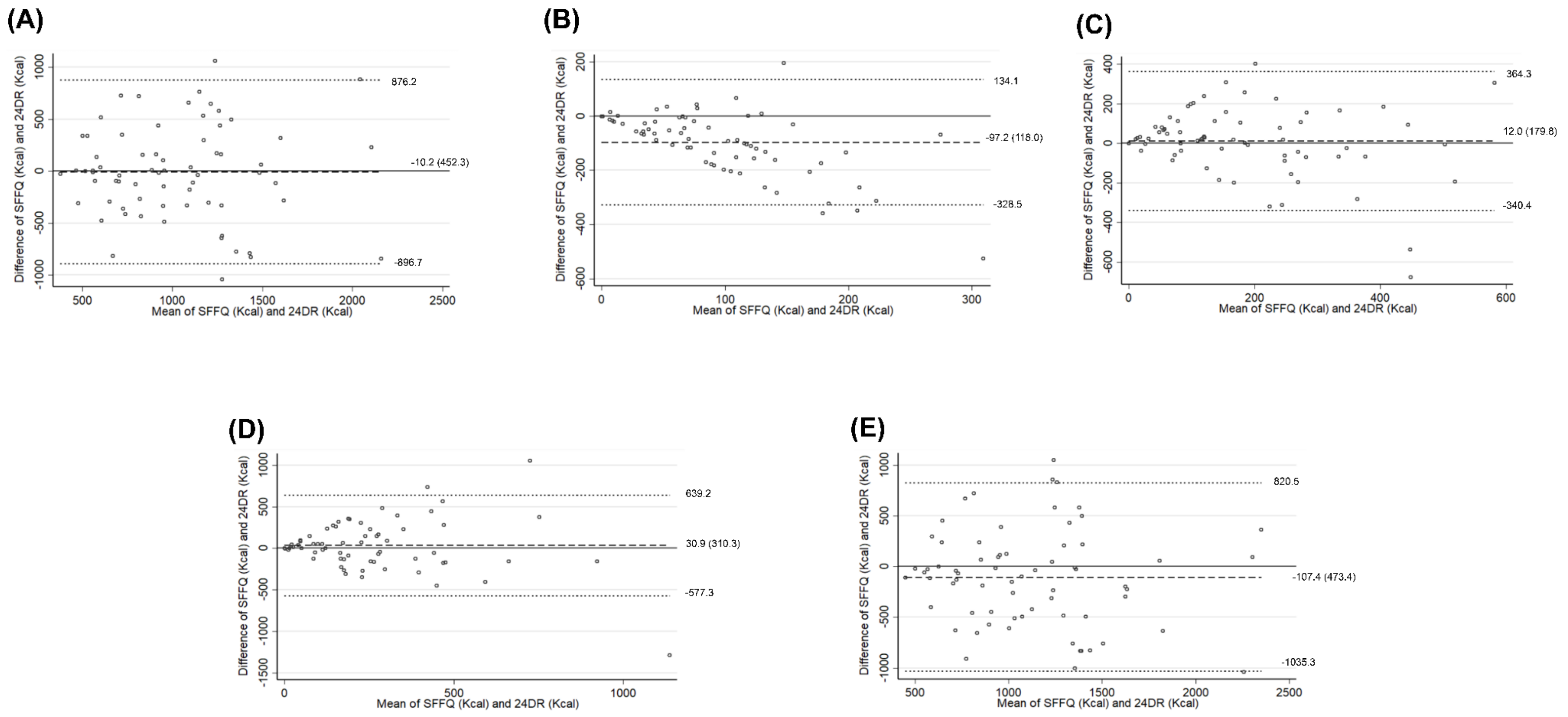

Supplementary figure 4. Older adults (≥60 y). Bland–Altman plots for % energy intake between the SFFQ and 24DRs: (A) Unprocessed and minimally processed foods group. (B) Processed culinary ingredients group. (C) Processed foods group. (D) Ultra-processed foods group. (E) Unprocessed and minimally processed foods group and Processed culinary ingredients group. Dotted lines represent 95% limits of agreement. Dashed line represents difference of mean.
